# Unexpected Relationships and Inbreeding in HapMap Phase III Populations

**DOI:** 10.1371/journal.pone.0049575

**Published:** 2012-11-19

**Authors:** Eric L. Stevens, Joseph D. Baugher, Matthew D. Shirley, Laurence P. Frelin, Jonathan Pevsner

**Affiliations:** 1 Predoctoral Program in Human Genetics, Johns Hopkins School of Medicine, Baltimore, Maryland, United States of America; 2 Program in Biochemistry, Cellular, and Molecular Biology, Johns Hopkins School of Medicine, Baltimore, Maryland, United States of America; 3 Department of Neurology, Hugo W. Moser Research Institute at Kennedy Krieger, Baltimore, Maryland, United States of America; 4 Department of Psychiatry and Behavioral Sciences, Johns Hopkins School of Medicine, Baltimore, Maryland, United States of America; University of Hong Kong, Hong Kong

## Abstract

Correct annotation of the genetic relationships between samples is essential for population genomic studies, which could be biased by errors or omissions. To this end, we used identity-by-state (IBS) and identity-by-descent (IBD) methods to assess genetic relatedness of individuals within HapMap phase III data. We analyzed data from 1,397 individuals across 11 ethnic populations. Our results support previous studies (Pemberton et al., 2010; Kyriazopoulou-Panagiotopoulou et al., 2011) assessing unknown relatedness present within this population. Additionally, we present evidence for 1,657 novel pairwise relationships across 9 populations. Surprisingly, significant Cotterman's coefficients of relatedness K1 (IBD1) values were detected between pairs of known parents. Furthermore, significant K2 (IBD2) values were detected in 32 previously annotated parent-child relationships. Consistent with a hypothesis of inbreeding, regions of homozygosity (ROH) were identified in the offspring of related parents, of which a subset overlapped those reported in previous studies (Gibson et al. 2010; Johnson et al. 2011). In total, we inferred 28 inbred individuals with ROH that overlapped areas of relatedness between the parents and/or IBD2 sharing at a different genomic locus between a child and a parent. Finally, 8 previously annotated parent-child relationships had unexpected K0 (IBD0) values (resulting from a chromosomal abnormality or genotype error), and 10 previously annotated second-degree relationships along with 38 other novel pairwise relationships had unexpected IBD2 (indicating two separate paths of recent ancestry). These newly described types of relatedness may impact the outcome of previous studies and should inform the design of future studies relying on the HapMap Phase III resource.

## Introduction

The International HapMap Project [1] identified common variation among single nucleotide polymorphisms (SNPs) within 11 distinct geographic groups (see Methods). The individuals were chosen to be representative of the genetic background within these populations. This information was used to identify patterns of linkage-disequilibrium and inform genome-wide association studies (GWAS) [1], [2], [3], contributing to our knowledge of genomic loci that have an influence on human health and disease [4]. Additional uses of HapMap data include GWAS of gene expression [5], [6], construction of haplotype maps [2], examination of joint allele frequency distributions [7], and investigation of regions that have undergone positive selection [8].

Since consanguinity occurs at different levels among inbred and outbred populations [9], [10], [11], and the offspring of related individuals may have regions of homozygosity due to autozygosity, tracts of homozygosity have been characterized within HapMap samples [12]. These results suggested that the majority of homozygosity found was due to decreased recombination at various genomic loci, with some individuals (NA12874, NA18992, and NA18987) highlighted as potentially having a greater degree of recent relatedness in their ancestry [12], [13]. Previous work has defined a region of homozygosity (ROH) as having a minimum length of either 500 kb or 1 Mb [14]; however, ROHs greater than or equal to 500 kb are relatively rare in outbred populations [15], [16]. Additional studies have identified regions having low recombination and long-haplotype sharing with evidence for distant sharing in several chromosomal regions among the different populations within HapMap [13], [17], [18].

Each HapMap population has been assumed to contain unrelated individuals unless otherwise annotated [1], [2], [3]. Recent work has established significant unannotated relatedness among 1,397 HapMap Phase III samples. In total, 604 unexpected, newly annotated relationships were established that consisted of identical, parent-child, full-sibling, second, third, and fourth-degree relationships [19], [20]. The majority of the relationships were from the Maasai in Kinyawa, Kenya (MKK), suggesting considerable background relatedness in that population [19]. Additional work has suggested the presence of further unannotated relatedness within HapMap Phase III [11], [18], [21].

In the present study, we report 1657 novel pairwise relationships across nine populations and validate previously reported relationships [19], [20] based on a method (kcoeff) [22] used to estimate Cotterman coefficients of relationship [23]. We present evidence of mislabeling among the annotated second-degree relationships (e.g. half-sibling as avuncular). In addition, some annotated relationships also had unexpected levels of IBD states for their relationship type. For example, there were parent-child relationships with IBD0 (indicating genotype miscall or deletion events involving expected IBD1) and IBD2 (possibly caused by a consanguineous union). We reconstructed a pedigree involving 171 individuals from MKK in which 149 were related to at least one other individual with an estimated K1 that exceeded 0.20. Also, we analyzed the amount of homozygosity in each sample, finding levels consistent with previous work [12], [13]. Finally, we present evidence for inbreeding in 28 individuals who resulted from a consanguineous union.

**Figure 1:**
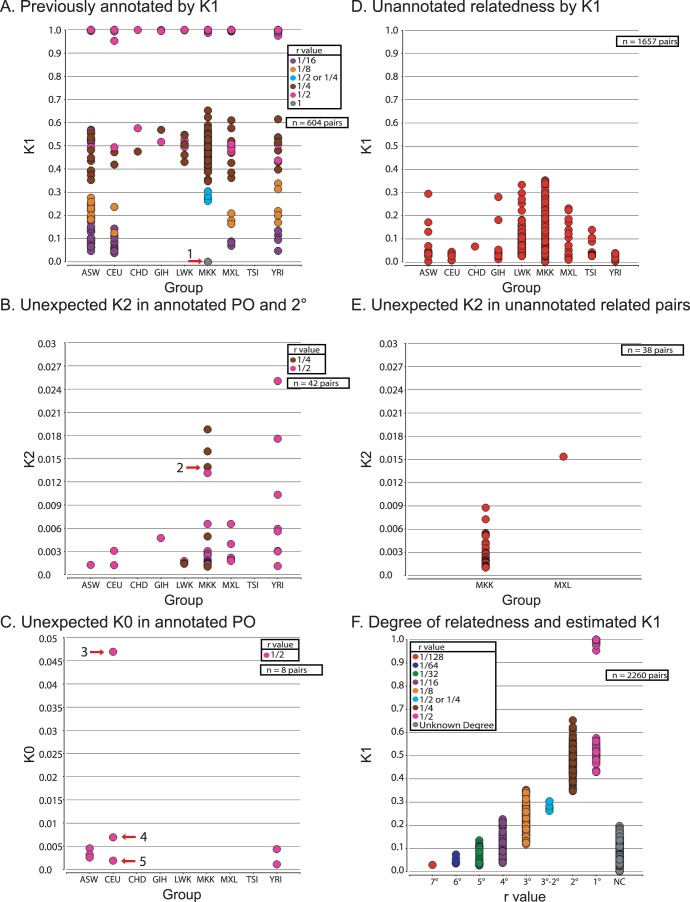
IBD estimates of previously annotated and novel relatedness in phase III HapMap. Each circle represents a pair of individuals with estimated Cotterman coefficients of relatedness K0, K1, and K2 (percent of the genome shared IBD0, IBD1, and IBD2). (A) Previously annotated relationships given by the International HapMap Consortium [2], Pemberton et al. [19], and Kyriazopoulou-Panagiotopoulou et al. [20] were plotted by group (x-axis) and K1 values (y-axis) and labeled by their degree of relationship. Arrow 1 corresponds to identical samples NA21737/NA21344. (B) Unexpected K2 values (y-axis) in previously annotated parent-child and second-degree relatedness for each group (x-axis). Only K2 values greater than 0.001 are shown. Arrow 2 corresponds to NA21362/NA21438. (C) Estimated IBD0 (y-axis) in previously annotated parent-child relationships for each group (x-axis). Only K0 values greater than 0.001 are shown. Arrows 3–5 highlight NA12874/NA12865, NA12889/NA12877, and NA10863/NA12234, respectively. Only K2 values greater than 0.001 are shown. (D) Novel relatedness between pairs of individuals separated by group (x-axis) and estimated K1 (y-axis). Only K1 values greater than 0.025 are shown. (E) Novel relatedness between pairs of individuals previously identified in Panel B for MKK and MXL (x-axis) with unexpected K2 (y-axis). (F) Inferred degrees of relationship (including those unable to be called; x-axis) plotted as a function of K1. All 2260 pairwise comparisons inferred to be related from any study (including this one) are shown, excluding identical samples. Note the overlap between percent of genome shared IBD1 and degree of relationship. Abbreviation: NC, no relationship called; r value, relatedness value.

## Results

### Unexpected Relationships in HapMap Phase III

We analyzed HapMap Phase III genotype data by computing all possible pairwise comparisons of autosomal IBD values for 1,397 individuals within each of the 11 geographic groups (n = 95,991 within-group comparisons). We plotted the within-group data for previously known relationships (n = 604) [2], [19], [20] (**Figure 1A**) and annotated by the degree of relationship assigned from previous studies. The relationship types clustered around their expected K1 values (estimated by kcoeff) descending from parent-child relationships near 1.0, to full-siblings, second, third, and fourth-degree relationships. An MZ twin pair from MKK (NA21344/NA21737) is highlighted having an expected K1 of zero (see arrow 1) and a K2 of 1.0 (data not shown).

**Table 1:** Previously annotated relationships and novel inference of relatedness.

	Previously Annotated						This Study							
Group	ID	PC	FS	1/4th	1/8th*	1/16th ∧	PC with IBD2	PC with IBD0	1/4th withIBD2	0.30≤K1<0.353	0.20≤K1<0.30	0.10≤K1<0.20	K1<0.10	0.025≤K1≤0.35 with IBD2
**ASW**	0	41	5	14	12	22	1	3	0	0	1	3	18	0
**CEU**	0	96	1	2	2	15	2	3	0	0	0	0	17	0
**CHB**	0	0	0	0	0	0	0	0	0	0	0	0	0	0
**CHD**	0	1	1	1	0	0	0	0	0	0	0	0	1	0
**GIH**	0	3	1	1	0	0	1	0	0	0	1	1	14	0
**JPT**	0	0	0	0	0	0	0	0	0	0	0	0	0	0
**LWK**	0	4	6	4	0	0	1	0	2	1	4	23	78	0
**MKK**	1	69	17	80	0	0	14	0	8	10	98	249	1104	37
**MXL**	0	56	3	10	3	3	5	0	0	0	2	5	7	1
**TSI**	0	0	0	0	0	0	0	0	0	0	0	2	11	0
**YRI**	0	110	3	7	6	4	8	2	0	0	0	0	7	0
**Total**	1	380	37	119	23	44	32	8	10	11	106	283	1257	38

Previously annotated relationships are reported for each of the 11 HapMap groups. ID/MZ, PC, FS, 1/4^th^ are as reported by individuals during data collection and Pemberton et al. 2010 [19]. 1/8^th^ and 1/16^th^ relationships are from [2], [20]. Novel inference of unexpected IBD sharing given the relationship type of known relationships is reported in columns denoted by PC with IBD2, PC with IBD0, and 1/4^th^ with IBD2. We report novel pairwise relatedness based on the amount of sharing detected IBD1 or IBD2 in the remaining columns. Abbreviations used: ID/MZ, identical or monozygotic samples; PC, parent-child; FS, full-sibling; 1/4^th^, second-degree relationships; 1/8^th^, third-degree relationships; and 1/16^th^, fourth-degree relationships.

We next analyzed those previously known parent-child (n = 32) and second-degree relationships (n = 10) for which we observed unexpected K2 sharing ≥0.001 (corresponding to autosomal loci cumulatively ≥3 Mb) **(** **Figure 1B**). The presence of appreciable IBD2 sharing between parent-child is indicative of potential relatedness between the parents, while IBD2 among second-degree relatives is caused by having two common ancestors of distinct lineage (bilineal). Relatedness between the parents could lead to inbreeding in the child as we describe below. Arrow 2 points to MKK pair NA21438/NA21362 with estimated K2 of 0.014. This pair was previously estimated by RELPAIR analysis as a full-sibling pair in 10/25 runs with 15/25 runs suggesting it to be a second-degree relative [19]; our data support the assignment of a second-degree relationship as full-siblings have an expected K2 of 0.25. This provides an example of the challenge of assigning a relationship type given atypical IBD sharing.

**Figure 2:**
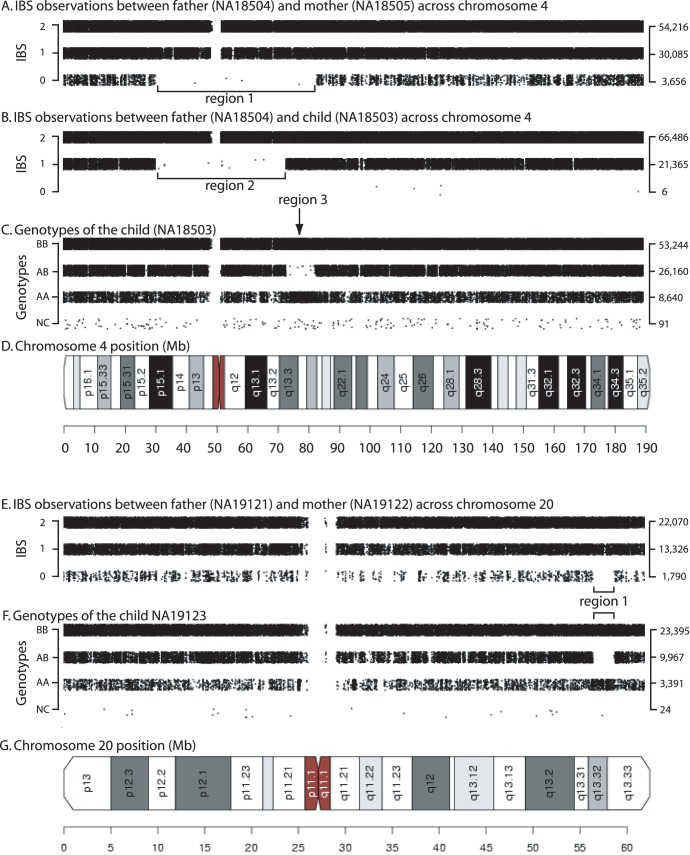
Evidence for consanguinity in HapMap Phase III individuals. Pairwise comparisons of IBS were plotted across a chromosome by position for pairs of individuals that had unexpected IBD1 and IBD2 for their relationship type. (A) IBS observations for two parents (YRI father/mother NA18504/NA18505) are shown for chromosome 4. Note region 1 which indicates an absence of IBS0 calls and inferred IBD1 status. (B) IBS measurements between father and son (NA18504/NA18503) are plotted for chromosome 4. Note region 2 in which there are few IBS0 and IBS1 calls thus implying IBD2 status. (C). Genotypes of the son (NA18503) are shown for chromosome 4. Note region 3 in which there is a lack of AB calls, aligning with region 1, thus indicating autozygosity. (D) Ideogram for chromosome 4. (E) IBS observations between two YRI parents (father/mother NA19121/NA19122) are plotted along chromosome 20. Note region 1 in which there is a lack of IBS0 calls indicating an IBD1 region. (F) Genotypes of the son (NA19123) are shown for chromosome 20. Note region 1 in which there are zero AB calls in the same region of IBD1 between the parents implying autozygosity in the child. (G) Ideogram for chromosome 20.

The observation of unexpected IBD0 in parent-child relationships may be indicative of an ROH resulting from a genotype miscall or hemizygous deletion. We observed 8 parent-child pairs with K0≥0.001 (**Figure 1C**). Arrow 3 points to CEU pair NA12874/NA12865 in which NA12874 is homozygous for almost the entirety of the q arm of chromosome 1 as reported previously [12] and as shown with SNPduo software which plots the identity-by-state (IBS) observations for a pair of individuals along with their respective genotypes on a per chromosome basis (**Figure S1A–D)** [24]. The K0 estimate of ∼0.047 is inflated due to the lack of heterozygous calls within this region of NA12874. This is a characteristic of the kcoeff program as previously noted [25]. An example of an apparent IBD0 event is illustrated in (**Figure S1E–H)**.

**Table 2:** Individuals inferred to be inbred.

ID	Group	ROH(Chr)	Total Mb	Total SNPs	Number of ROHs	Average Size(Mb)	Average SNPs	Parents Related	IBD2 with Parent	Comment
NA10852	CEU	2, 4, 8, 12, 13	35.2	19329	5	7.0	3866	NA12046absent	NA12045	Inbred
NA12766	CEU	No ROH	0.0	0	0	0.0	0	NA12776/NA12775	NA12775	Not inbred
NA12832	CEU	2, 4, 6, 8	18.4	7446	7	2.6	1064	NA12842/NA12843	No	Inbred
NA18503	YRI	1, 4	15.6	6264	2	7.8	3132	NA18504/NA18505	NA18504/NA18505	Inbred
NA19123	YRI	3, 6, 8, 17, 20	12.8	8415	5	2.6	1683	NA19121/NA19122	NA19121/NA19122	Inbred
NA19173	YRI	No ROH	0.0	0	0	0.0	0	NA19172/NA19171	NA19172	Not inbred
NA19186	YRI	12	2.3	739	1	2.3	739	NA19184/NA19185	NA19185	Inbred
NA19224	YRI	8, 10,12, 20	29.6	15015	4	7.4	3754	NA19226/NA19225	NA19226/NA19225	Inbred
NA19434	LWK	1, 10, 11	9.6	4881	3	3.2	1627	Single parent	No	Sibling to NA19444
NA19444	LWK	7	2.4	1339	1		1339	Single parent	NA19432	Sibling to NA19434
NA19650	MXL	1, 7, 8, 11, 12, 14	24.5	10059	7	3.5	1437	NA19648absent	NA19649	Inbred
NA19653	MXL	3, 4, 6, 11, 16	30.9	13656	8	3.9	1707	NA19651/NA19652	NA19651/NA19652	Inbred
NA19775	MXL	6,7,17	9.8	5105	3	3.3	1702	NA19773/NA19774	NA19774	Inbred
NA19787	MXL	2, 3, 4, 6,9, 11	22.4	8596	7	3.2	1228	NA19785/NA19786	NA19785	Inbred
NA19983	ASW	No ROH	0.0	0	0	0.0	0	NA19982/NA19713	NA19982	Not inbred
NA20900	GIH	2, 3, 6, 8	15.3	7103	4	3.8	1776	NA20882/NA20891	No	Inbred
NA20909	GIH	1, 12, 17	10.3	4677	4	2.6	1169	Single parent	NA20909	Unknown PO order
NA20910	GIH	2, 3, 17	31.6	17742	4	7.9	4436	Single parent	NA20910	Unknown PO order
NA21311	MKK	6	24.6	11779	1	24.6	11779	Single parent	NA21314	Inbred
NA21317	MKK	2, 14	7.0	3569	2	3.5	1785	NA21316/NA21580	NA21316	Inbred
NA21361	MKK	No ROH	0.0	0	0	0.0	0	NA21359/NA21360	NA21360	Not inbred
NA21384	MKK	5, 17	8.7	3536	2	4.3	1768	NA21387/NA21388	NA21388	Inbred
NA21389	MKK	No ROH	0.0	0	0	0.0	0	NA21387/NA21388	NA21387	Not inbred
NA21401	MKK	3, 8, 11,12, 15	19.4	7817	6	3.2	1303	NA21400/NA21399	No	Inbred
NA21425	MKK	2, 12, 16	28.4	13256	4	7.1	3314	NA21423/NA21424	NA21423/NA21424	Inbred
NA21439	MKK	2, 13	7.6	4378	3	2.5	1459	NA21438/NA21447	NA21447	Inbred
NA21475	MKK	7, 13	6.0	3072	2	3.0	1536	NA21488/NA21489	NA21488	Inbred
NA21477	MKK	6	2.1	997	1	2.1	997	NA21476/NA21475	NA21475	Inbred
NA21490	MKK	1, 6,11, 13	12.7	9891	5	2.5	1978	NA21488/NA21489	No	Inbred
NA21514	MKK	2	5.3	3036	2	2.6	1518	NA21513/NA21512	NA21513	Inbred
NA21527	MKK	7	4.8	2040	2	2.4	1020	NA21526/NA21583	NA21526	Inbred
NA21601	MKK	6	3.2	1323	1	3.2	1323	NA21600/NA21599	NA21600	Inbred
NA21608	MKK	1, 7	9.6	3483	2	4.8	1742	NA21615/NA21614	No	Inbred

Individuals listed as inbred are annotated with the following information: Group; ROH (Chr), chromosomes harboring regions of homozygosity (absence of AB calls); Total Mb, total length of all ROHs ≥2 Mb and ≥400 SNPs; Total SNPs, number of SNPs present in all ROHs; Number of ROHs; Average Size (Mb), Average lengths of ROHs; Average SNPs, Average number of SNPs per ROH; Parents Related, were the individuals assigned an inbred status because of IBD1 detected between the parents in a region where the child is homozygous (placement of parents within the column indicates presence of IBD1); IBD2 with Parent, IBD2 detected between the child and a parent indicates the parents are related (individuals placed in this column represent the parent(s) with whom the child has IBD2; Comment; additional information relevant to the inbred individual. Note that individuals who have relatedness between the parents are not inferred to be inbred if there are no reported regions of homozygosity in that individual that overlap an inferred region of IBD1 between his/her parents.

The occurrence of K1 between two people provides evidence for relatedness, particularly if the amount is sufficiently high (we applied a cutoff value of 0.025). We detected 1657 pairwise relationships involving individuals previously annotated as unrelated (shown by group in **Figure 1D**). This relatedness was confirmed using SNPduo analysis to observe autosomal regions (≥10 Mb) lacking IBS0. Four pairwise comparisons (NA19763/NA19670, NA19656/NA19681, NA21090/NA21109, and NA21125/NA21098) all had K1 values over 0.025 (with 0.028 being the highest) but we did not annotate these as related since SNPduo analysis did not reveal a region indicating IBD1. This is presumably due to multiple regions of low variability (conserved haplotypes representing ancestral sharing) between individuals that result in elevated K1 values for recently unrelated individuals.

**Table 3:** Corrected relationships from Pemberton et al. 2010.

IID1	IID2	Group	k0	k1	k2	Inferred	Annotated	Reason	Comments
NA12874	NA12865	CEU	0.0470	0.9530	0.0000	PC_IBD0	PC	IBD0 affectsRELPAIR PO	3/25 panels were GG
NA12877	NA12889	CEU	0.0069	0.9931	0.0000	PC_IBD0	PC	IBD0 affectsRELPAIR PO	1/25 panels were GG
NA19381	NA19382	LWK	0.0000	1.0000	0.0000	PC	PC	NA19382 is the Father	Annotated as unknown PO
NA21300	NA21520	MKK	0.3475	0.6525	0.0000	HS	GG	Scenario 3, 5	
NA21300	NA21613	MKK	0.5723	0.4277	0.0000	HS	GG	Scenario 3, 5	
NA21312	NA21370	MKK	0.5377	0.4623	0.0000	HS	2°	Scenario 1, 3	
NA21312	NA21617	MKK	0.4541	0.5459	0.0000	AV	2°	Scenario 1	NA21617 is AV
NA21320	NA21311	MKK	0.4788	0.5205	0.0008	HS	GG	Scenario 3	
NA21320	NA21312	MKK	0.3951	0.5890	0.0159	HS_IBD2	GG	Scenario 3	
NA21362	NA21438	MKK	0.5899	0.3962	0.0139	AV_IBD2 ∧	HS	Scenario 5 IBD2 affects RELPAIR FS	10/25 Panels were FS
NA21378	NA21448	MKK	0.5163	0.4837	0.0000	HS	GG	Scenario 5	
NA21408	NA21450	MKK	0.4976	0.5024	0.0000	HS	2°	Scenario 3	
NA21414	NA21351	MKK	0.5171	0.4829	0.0000	AV	2°	Scenario 1, 4	NA21414 is AV
NA21414	NA21352	MKK	0.4911	0.5089	0.0000	AV	2°	Scenario 1, 4	NA21414 is AV
NA21415	NA21363	MKK	0.0000	1.0000	0.0000	PC	PC	NA21415 is the Mother	Annotated as unknown PO
NA21420	NA21524	MKK	0.6962	0.3036	0.0001	3°	2°		Pemberton et al. were conservative
NA21421	NA21485	MKK	0.5357	0.4635	0.0008	HS	2°	Scenario 4	
NA21423	NA21447	MKK	0.5757	0.4243	0.0000	HS	AV	Scenario 3	
NA21435	NA21647	MKK	0.6530	0.3470	0.0000	HS	2°	Scenario 4	
NA21448	NA21493	MKK	0.5626	0.4374	0.0000	HS	GG	Scenario 4	
NA21453	NA21378	MKK	0.4467	0.5533	0.0000	HS	2°	Scenario 3	
NA21453	NA21450	MKK	0.5657	0.4343	0.0000	HS	AV	Scenario 3	
NA21453	NA21493	MKK	0.4504	0.5496	0.0000	HS	2°	Scenario 3	
NA21488	NA21478	MKK	0.5812	0.4188	0.0000	AV	2°	Scenario 1*	NA21488 is AV
NA21488	NA21485	MKK	0.5085	0.4908	0.0008	AV	2°	Scenario 1*	NA21488 is AV
NA21519	NA21316	MKK	0.4492	0.5508	0.0000	HS	GG	Scenario 4	
NA21519	NA21318	MKK	0.5562	0.4423	0.0015	HS_IBD2	GG	Scenario 4	
NA21519	NA21635	MKK	0.7278	0.2722	0.0000	3°	GG		Pemberton et al. were conservative
NA21575	NA21574	MKK	0.0000	1.0000	0.0000	PC	PC	NA21574 is the Mother	Annotated as unknown PO
NA21576	NA21357	MKK	0.7379	0.2621	0.0000	3°	GG		Pemberton et al. were conservative
NA21576	NA21509	MKK	0.7162	0.2838	0.0000	3°	GG		Pemberton et al. were conservative
NA21599	NA21521	MKK	0.5052	0.4949	0.0000	HS	2°	Scenario 4	
NA21617	NA21370	MKK	0.5083	0.4917	0.0000	AV	2°	Scenario 1	NA21617 is AV
NA21617	NA21520	MKK	0.4535	0.5277	0.0188	HS_IBD2	2°	Scenario 4	
NA21617	NA21613	MKK	0.5319	0.4632	0.0049	HS_IBD2	2°	Scenario 4	
NA21634	NA21435	MKK	0.5312	0.4688	0.0000	HS	2°	Scenario 4	
NA21634	NA21647	MKK	0.5634	0.4366	0.0001	HS	2°	Scenario 4	
NA21686	NA21520	MKK	0.4843	0.5157	0.0000	HS	AV	Scenario 4	
NA21686	NA21613	MKK	0.5776	0.4224	0.0000	HS	AV	Scenario 4	
NA21320	NA21314	MKK	0.0000	1.0000	0.0000	PC	PC	NA21314 is the Father	NA21320 was annotated as the Mother
NA21678	NA21519	MKK	0.7294	0.2706	0.0000	3°	2°		Pemberton et al. were conservative

Previously designated relationships for annotated pairs are reassigned based on pedigree reconstruction methods or IBD analysis. Note that certain relationships annotated correctly by previous studies (and Pemberton et al. 2010 [19]) are included because of the addition of further information. For example, NA12874 and NA12865 were correctly assigned a parent-child relationship but we amend that to parent-child_IBD0 based on the presence of apparent IBD0 between them. Scenarios used to prove or rule out a relationship type are provided in the Supplemental Method File. Abbreviations used: Inferred, our annotation for a given pairwise comparison; PO, parent-child; AV, avuncular; GG, grandparent-grandchild; HS, half-sibling.

Thirty-eight of those newly annotated pairs had IBD2 estimates ≥0.001 (**Figure 1E**). All but one (MXL pair NA19657/NA19787) were from the MKK population. This result is expected since the large amount of relatedness within MKK would increase the probability that two individuals shared at least two independent common ancestors (discussed in detail below). We generated a complete list of IBD estimates for 2261 pairwise comparisons that have evidence of recent ancestry (604 previously annotated plus 1657 newly reported) (**Table S1)**. Out of the 1,397 individuals involved in HapMap Phase III, 785 are related to at least one other individual (with a K1 greater than 0.025, except between parents of an inbred child).

**Figure 3:**
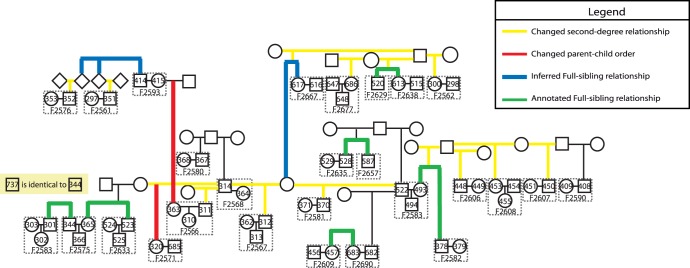
Reconstruction of a partial pedigree from the MKK group. We analyzed MKK genotype data using IBD analysis and inferred the familial relationships of 61 individuals with 46 being related to at least 1 other person. This graph contains relationships constructed from second-degree, full-sibling, parent-child, and identical relationships (with the exception of NA21352 and NA21351 who are inferred to be first-cousins based on their second-degree relationship to NA21414; see top left of figure). All indicated relationships are based on previous analysis (siblings: thick green lines), previous annotation (family trios; family ID), and inferred analyses (sibling relationships, thick blue lines; corrected parent-child orientation, thick red lines; corrections made to annotated relationships, thick yellow lines; other familial relationships; thin black lines). Dashed rectangles indicate family units annotated by the HapMap project at the Coriell website. F indicates family identifier (e.g. F2654). Individual identifiers are shown as the last three digits of NA21xxx (e.g. 353 at the upper left of the figure corresponds to individual NA21353). All IBD information is given in Table S1. Note that several individuals who are part of MKK (e.g. NA12310 in family 2566) and for whom cell lines were created did not have SNP data as part of the HapMap Phase III release.

Estimating the degree of relationship for a given pair of individuals is unequivocal for identical samples that share 100% IBD2, parent-child pairs that share 100% IBD1, and full-sibling pairs that share 25% IBD0, 50% IBD1, and 25% IBD2. Past research has shown that there is variation among percent of the genome shared for full-siblings (e.g. IBD1 with ranges of 0.38–0.62) [26], [27] and we agree with this range. Past research has also shown there is virtually no overlap between estimated IBD1 sharing between 1/4^th^ and 1/8^th^ relationships but there is considerable overlap between third, fourth, fifth-degree relationships and higher [28]. We report total numbers of annotated and newly reported related pairwise comparisons by group and K1 and K2 levels in **Table 1**. The majority of the relationships are within MKK (see below).

We plotted the degrees of relatedness with respect to the distribution of K1 value (**Figure 1F**). We started with parent-child and full-sibling relationships (as well as second-degree relationships either previously known [2], [19] or ones with K1 values >0.35) and annotated as many pairwise comparisons by degree of relatedness as possible. We also labeled third and fourth-degree relationships from previous publications but cannot support all those relationships [20]. We were able to annotate seventh-degree relationships within MKK due to the nature of extensive sharing and building off full-sibling, parent-child, and second-degree relationships; however, the majority of pairwise comparisons with K1 values indicative of relatedness were not assigned a degree of relationship. The figure illustrates the overlap between second-degree and third-degree relationships. The K1 distribution for second-degree relationships spans 0.3–0.7 as measured in annotated pedigrees [25], while third-degree relationships may have values as high as 0.35. It is also apparent that the K1 overlap between degrees of relatedness increases as the number of generations to a common ancestor becomes greater.

We identified 28 individuals who were inferred to be inbred based upon a ROH overlapping with a region of unexpected IBD1 between their parents. Parent-child relationships with IBD2 (**Figure 1B**) provided further support for consanguineous unions but do not overlap ROH. The ROH that are inferred to be autozygous segments in an inbred individual are provided in **Table S2** (individual regions) and **Table S3** (total Mb of homozygosity). We identified five additional individuals with related parents but no autozygous segments that overlapped regions of IBD1 between the parents. Following previous work [25], we used SNPduo to visualize inferred regions of IBD1 (**Figure 2A**; see region 1) that had an absence of IBS0 calls between two parents (NA18504 and NA18505) overlapping a region of IBD2 (**Figure 2B**; see region 2) between NA18504 and the child (NA18503) along chromosome 4 (**Figure 2D**). Region 1 also overlapped a region (**Figure 2C**; see region 3) of homozygosity in the child that indicates inheritance of the same allele from both parents. A second example is provided in **Figure 2E–G** for parents (NA19121 and NA19122) and the child (NA19123) across chromosome 20. Examples of IBD2 sharing among second-degree relationships are provided in **Figure S2**.

We report all inbred individuals in **Table 2**. We include additional information on the extent of homozygosity due to autozygosity, the number of regions, chromosomes affected, and the reason we report inbreeding (i.e. parents were related and/or IBD2 was detected in a parent-child relationship). Finally, some individuals are not reported to have autozygous segments (and are not inferred to be inbred) but the parents of these individuals are related or the child shares IBD2 with a parent. IBD1 estimates between the parents of inbred individuals and IBD1 estimates between the parents or IBD2 estimates between a parent and a child who is not inbred (see above) are presented in **Table S1**.

Using logic-based methodologies (see Methods), we reconstructed 34 relationships and provide evidence for corrected annotations for pairwise comparisons in **Table 3**. For example, we were able to infer avuncular status of NA21617 to NA21370 and NA21312 by finding regions where NA21617 was related to both individuals at the same locus while the inferred half-siblings were unrelated to each other at that position (see Methods). Using estimates of IBD, we present additional evidence that atypical IBD sharing can affect parent-child relationships (e.g. small amounts of IBD0 can result in RELPAIR inferring grandparent/grandchild relationships in a small percentage of runs) or second-degree relationships (e.g. small amounts of IBD2 can result in RELPAIR inferring full sibling relationships) (see [25]). Pemberton et al. proposed the creation of a dataset of 1161 individuals having no parent-child or full sibling pairs (“HAP1161”), as well as HAP1117 also having no second-degree relative pairs. Their analysis concluded that five pairwise comparisons likely involved 1/8^th^ relationships. However, in an effort to treat the data analysis conservatively, they classified these as 1/4^th^ related and removed them from HAP1117 [19]. The present study confirms that these are likely 1/8^th^ relationships (**Table 3**), supported by the estimated amount of K1 (**Figure 1A, F**; see blue circles). We further identified a parent-child relationship (NA21737/NA21366) and a full-sibling relationship (NA21737/NA21301) in HAP1161 that should be excluded.

Additionally, we were able to infer the pedigree structure for a subset of individuals in MKK. We present a pedigree of 61 individuals in which 46 are related to at least one other person to show the extent of familial sharing present (**Figure 3**). We present the full pedigree of 171 individuals in which 138 are related to at least one other person for K1 values exceeding 0.20 (**Figure S3**).

We also detected unusual K2 values indicating that a few megabases (K0 ∼ 0.001) were shared among unrelated individuals (**Figure S4**). Subsequent analysis with SNPduo highlighted two regions (6p22.1 and 11p11.2) in which IBD1, IBD2, and/or ROH were seen in the majority of pairwise comparisons with elevated K2 levels (data not shown). Previous studies have found considerable homozygosity and allele sharing at these loci due to the presence of long haplotypes that are conserved [13], [17].

## Discussion

Our results provide a detailed and definitive estimate of all recent ancestry within HapMap Phase III in which the estimated level of IBD1 exceeds 0.025 (slightly less than the expected amount of relatedness for seventh-degree relatives). We identified an additional 1657 relationships representing nine of the eleven ethnic populations: ASW, CEU, CHD, GIH, LWK, MKK, MXL, TSI, and YRI. Furthermore, we present evidence for reassigning relationship type to 30 second-degree relationships (e.g. half-sibling to avuncular), assigning 32 previously annotated parent-child relationships with unexpected IBD2, assigning 8 previously annotated parent-child relationships with unexpected IBD0, and assigning 10 previously annotated second-degree relationships with unexpected IBD2 [2], [19].

In addition 28 individuals are inferred to be inbred based upon relatedness between the parents and/or IBD2 between a parent and the child coinciding with ROH in the child. Five additional individuals had related parents but no ROH due to autozygosity. Since our methods of inferring inbreeding in a child required the presence of a parent to facilitate identification and previous publications have established ROH present within various samples [12], [13], it is possible that many more inbred individuals exist within the different HapMap populations. In fact, we also uncovered two genomic regions on chromosomes 6 and 11 that confer low levels of inferred IBD2 sharing (as well as extended tracts of homozygosity) that were previously identified as being within regions of low recombination [17], [18]. Taken together, these results suggest that distant relatedness is shared both within and between populations.

The HapMap collection has served as a primer for understanding common genetic variation both within and between populations. HapMap samples are also a part of the 1000 Genomes Project, which seeks to identify and characterize 95% of alleles having a frequency of 1% or higher in genomic regions accessible to high-throughput sequencing technologies in various populations of the world [29]. Central to this work is the inclusion of unrelated individuals to accurately estimate appropriate levels of variation. These collections were used to map structural variations [30], uncover areas of frequent recombination events [31], and look for evidence for or against classic selective sweeps in the human genome [32].

Since all members of the CEU population of HapMap overlap with the Centre d’Étude du Polymorphisme Humain (CEPH) pedigrees [2], [33], [34], there exists a set of pedigrees and individuals in other studies that are inferred to be related. Previous work has already addressed the issue of relatedness and consanguinity within subsets of the CEPH collection [25], [35], [36]. These results could extend to previous work, such as research into the inheritance of gene expression in which the relatedness was not explicitly accounted for [37], [38], [39]. With a history of uncovering unannotated relatedness in datasets that have been used extensively throughout the literature by others and by us [19], [20], [22], [25], [36], we recommend more stringent measures of quality control as part of the analysis of experiments with outcomes which may be sensitive to unannotated relatedness.

## Materials and Methods

### HapMap Genotype Data

We obtained HapMap Phase III genotype data (hapmap3_r3/deposited 12 February, 2010 and downloaded March 20, 2010). The data were from 1,397 individuals representing 11 distinct geographic groups: African ancestry in Southwest USA (ASW; n = 87 individuals); Utah residents with Northern and Western European ancestry from the CEPH collection (CEU; n = 165); Han Chinese in Beijing, China (CHB; n = 137); Chinese in Metropolitan Denver, Colorado (CHD; n = 109); Gujarati Indians in Houston, Texas (GIH; n = 101); Japanese in Tokyo, Japan (JPT; n = 113); Luhya in Webuye, Kenya (LWK; n = 110); Maasai in Kinyawa, Kenya (MKK; n = 184); Mexican ancestry in Los Angeles, California (MXL; n = 86); Tuscans in Italy (TSI; n = 102); and Yoruba in Ibadan, Nigeria (YRI; n = 203). We only included data from autosomes and removed × chromosome and mitochondrial SNPs. We used PLINK [40] to convert a "ped" file from nucleotide to numeric format.

### IBS and IBD Analyses

The genotype data was analyzed for IBD using kcoeff software [22] that estimates the percent of genome shared IBD0 (K0), IBD1 (K1), and IBD2 (K2) (i.e. Cotterman coefficients of relatedness k0, k1, and k2). kcoeff removed all SNPs that were concordant homozygotes resulting in an average of 712,112 informative SNPs remaining for each pairwise comparison. We estimated IBD with a window size of 500 informative SNPs. For the 1,397 individuals from 11 different ethnic populations, we performed a total of 975,106 pairwise comparisons including 95,991 within-group comparisons. We analyzed IBS with SNPduo (a web-based program that generates plots and tables of IBS sharing across chromosomes) [24]. SNPduo++[24] was used to analyze all 95,991 within-group pairwise comparisons between the 1,397 samples) and to generate IBS2*_ratio values of [IBS2*/(IBS0+IBS2*)], where IBS2* denotes AB/AB genotypes [22], [41]. Regions of IBD are visually inferred from figures that plot IBS observations between individuals and are used throughout. Additionally, regions of IBD are calculated from kcoeff given SNP data which analyses regions of IBS between two individuals over contiguous regions throughout the genome.

### Homozygosity Analyses

We used a previously developed algorithm [25] to identify regions of homozygosity for every individual in a population. Minimal regions were defined as those being ≥2 Mb and ≥400 SNPs.

### Homozygosity and Distant IBD

Our kcoeff IBD method was robust for inferring relationships with an estimated K1≥0.025. We previously established a method for comparing regions of homozygosity in offspring to possible regions of IBD1 between the parents indicating when the homozygosity is due to autozygosity [25]. We modified this approach to include the minimum regions of homozygosity ≥2 Mb and ≥400 SNPs. Copy number information was not used to discriminate those ROH that result from a hemizygous deletion. A ROH in a child overlapping a region of IBD1 between the parents is evidence of inbreeding (as given in **Table 2**). Since parents were available for a small percentage of individuals, the majority of the ROH reported in **Tables S2–3** could be due to a hemizygous deletion or autozygosity.

### Reconstruction of Pedigrees

Inferring the degree of relationship allows for a potential classification of the type of relationship. For example, a pair of individuals inferred to be second-degree relatives could be inferred to be half-siblings, as opposed to grandparent-grandchild or avuncular. We present a method for reconstructing second-degree and third-degree relationships based on multiple pairwise comparisons. This approach requires specific information based on how alleles are shared. We provide five scenarios (as seen in **Table 3**) for classifying second-degree relationships: Scenario 1, inferring an avuncular (AV) relationship to two half-siblings (HS); Scenario 2, inferring an AV relationship to two full-siblings (FS); Scenario 3, inferring HS; Scenario 4, inferring a third or fourth-degree relationship; and Scenario 5, ruling out specific types of relationships. These methods are described in detail in the supporting information as well as **Figures S5–11** and **Table S4**. The majority of this method was applied to the MKK population and a section of the reconstructed pedigree is presented in **Figure 3**. The full pedigree is contained in **Figure S3** and links all relationships with a K1 value greater than 0.20. Note that some of the relationships are indicated by the estimated degree of relationship as full reconstruction of relationship type is not possible without more information.

## Supporting Information

Figure S1 **Evidence for apparent IBD0 sharing between previously annotated parent-child relationships.** For two pairs of related individuals who were previously annotated parent-child, we show IBD0 sharing across various chromosomes as provided by SNPduo analysis. For each pairwise comparison the three tracks are IBS0, IBS1, and IBS2. We also show the genotypes of the individuals, which indicate the individual who has the genotype profile that leads to the measured IBS0. For each individual the genotype tracks are BB, AB, AA, and NC (missing genotype). (A) Previously annotated parent-child relationship between CEU members NA12874 (maternal grandfather) and NA12865 (mother) has apparent IBD0 across chromosome 1. (B) Genotypes of NA12874, which reveal considerable homozygosity across the q arm. Note the IBS0 in this region. (C) Genotypes of NA12865, which are normal across the entire chromosome. (D) Ideogram of chromosome 1. (E) Previously annotated parent-child relationship between YRI members NA18498 (father) and NA18497 (son) across chromosome 1 has apparent IBD0. (F) Genotypes of NA18497 in which a region of dense NCs overlaps a region lacking AB calls in the same region of IBS0 between the parent-child relationship. (G) Genotypes of NA18498, which are normal across the entire chromosome. (H) Ideogram of chromosome 1.
(EPS)

Figure S2 **Evidence for IBD2 sharing between individuals.** For four pairs of related individuals who were annotated as related (either previously or in this study), we show IBD2 sharing across various chromosomes as provided by SNPduo analysis. For each pairwise comparison the three tracks are IBS0, IBS1, and IBS2. (A) Previously annotated second-degree relationship between LWK members NA19334 and NA19313 has unexpected IBD2 sharing on chromosome 19. (B) Previously annotated second-degree relationship (inferred by us to be avuncular) between MKK members NA21362 and NA21438 has IBD2 sharing across large regions of chromosome 1. Note that this pair had 10/15 full-sibling annotations given by RELPAIR from Pemberton et al [19]. (C) Newly annotated relationship of an unknown degree between MXL members NA19657 (mother of family M007) and NA19787 (son of family M032) has IBD2 sharing on chromosome 9. (D) Previously annotated avuncular relationship between LWK members NA19443 and NA19469 has IBD2 sharing on chromosome 4.
(EPS)

Figure S3 **Reconstruction of the full MKK pedigree.** We analyzed MKK genotype data using IBD analysis and inferred the familial relationships of 171 individuals with 149 being related to at least 1 other person. This graph contains all relationships with a K1 value greater than or equal to 0.20. All indicated relationships are based on previous analysis (siblings: thick green lines), previous annotation (family trios; family ID), and inferred analyses (sibling relationships, thick blue lines; corrected parent-child orientation, thick red lines; other familial relationships; thin black lines). Note that some relationships could not be resolved with certainty and the estimated degree of relationship is indicated on the line between them (with an *). Also note that some individuals are related through multiple nodes and are represented by unique colors. For example, 647 (NA21647) is represented in two places and is highlighted by a light blue background. Dashed rectangles indicate family units annotated by the HapMap project at the Coriell website. F indicates family identifier (e.g. F2654). Individual identifiers are shown as the last three digits of NA21xxx (e.g. 382 at the upper left of the figure corresponds to individual NA21382). All IBD information is given in Table S1. A subset of this pedigree is presented in Figure 3.
(EPS)

Figure S4 **Evidence for Haplotype sharing.** We analyzed HapMap genotype data using IBS (IBS2*_ratio from SNPduo++ [22], [24] software) and IBD (kcoeff software) for every HapMap Phase III within-group comparison. Full-sibling, parent-child, and annotated second-degree relationships were removed and the IBS2*_ratio for every remaining pairwise comparison was plotted on the x-axis with kcoeff’s estimate of K2 (estimated Cotterman coefficient for percent of the genome shared IBD2) on the y-axis. Note that the elevated K2 levels seen when samples have an IBS2*_ratio of 2/3 (nominally associated with unrelated pairs of individuals). This bump represents distant sharing of long haplotypes on chromosomes 6 and 11.
(EPS)

Figure S5 **Determination of avuncular relationship given two half-siblings or two full-siblings.** A pedigree is shown in panel A that provides an example for determining if two individuals who are half-siblings (1 and 2) are in an avuncular relationship with a third individual (3) by analyzing haplotype sharing on each chromosome. Panel B provides an example of determining an avuncular relationship between two full-siblings (1 and 2) and a third individual (3) who is the uncle (or aunt). Note the colored blocks by each individual are an ideogram of four 10 Mb haplotype blocks. Also note that since siblings are expected to share 25% of the genome IBD2, the sibling of individual 3 is able to substitute his/her genotypes in these regions to track what alleles were inherited by each child (individuals 1 and 2) and thus shared IBD1 with individual 3.
(EPS)

Figure S6 **Determination of a third-degree relationship given three related individuals and two second-degree relationships.** Each panel (A–E) represents one of the five possible pedigrees illustrating three related individuals between which there are two second-degree and one third-degree relationships. The colored blocks by each individual are an ideogram of four 10 Mb haplotype blocks. Note that the regions shared between individuals 1 and 3 are not always dependent on what individual 2 shares with them (see e.g. regions with a +). Some of the regions shared between individuals 1 and 3 are determined by the regions shared between individuals 1 and 2 and are labeled with an *. The use of # indicates a shared allele among individuals 1 and 2 or 2 and 3. Pedigrees A and B are indistinguishable from each other, but can be distinguished from pedigrees C–E. Pedigrees C–E can be distinguished from each other according to the following: C: All three individuals may be related to the other individuals at a position where the other individuals are unrelated to each other (opposite inheritance) and individuals 1 and 2 share IBD2 at another location; D: All three individuals may be related to the other individuals at a position where the other individuals are unrelated to each other (opposite inheritance); E: individual 1 and 2 may be related to the other individuals at a position where the other individuals are unrelated to each other (opposite inheritance) and individuals 1 and 2 share IBD2 at another location. Note that while individuals 1 and 3 can be inferred to be first-cousins in panels A and B, individual 2 could be in a grandparental or avuncular relationship to them.
(EPS)

Figure S7 **Determination of a fourth-degree relationship given three related individuals and two second-degree relationships.** Each panel (A–E) represents one of the five possible pedigrees illustrating three related individuals between whom there are two second-degree and one fourth-degree relationships. The colored blocks by each individual are an ideogram of four 10 Mb haplotype blocks. The use of * and # is the same as in **Figure 3**. The five pedigrees are indistinguishable from each other based on genetic data alone. Note that while individual 2 can be established as a grandparent in each pedigree, individuals 1 and 3 are interchangeable with each other.
(EPS)

Figure S8 **Evidence to support a known grandparent-grandchild relationship.** A pedigree is shown in panel A that highlights a known grandparent-child relationship between individuals 1 and 2, and their relationship to individual 3. SNPduo images along chromosome 7 show IBS observations between individuals 2 and 3 (panel B) and individuals 1 and 3 (panel C). Note that individual 1 only shares segments IBD1 with individual 3 that individual 2 shares IBD1 with individual 3. Panel D provides an ideogram for chromosome 7. Note the boxed regions indicating sharing of the same segment in all three individuals.
(EPS)

Figure S9 **Determination of avuncular relationship given two half-siblings.** A pedigree is shown highlighting a known avuncular relationship (individual 3) to two half-siblings (panel A; individuals 1 and 2). Panel B is a pediSNP image in which the avuncular individual’s genotype (3) is compared to the genotypes of the half-siblings (1 and 2) along chromosome 7. Note that the boxed region with asterisks highlights an opposite inheritance region. SNPduo images show the pairwise IBS observations between individuals 1 and 3 (panel C), 2 and 3 (panel D), and 1 and 2 (panel E). Note that individuals 1 and 2 are both related to individual 3 in the boxed region but are unrelated to each other. Panel F provides an ideogram for chromosome 7.
(EPS)

Figure S10 **Determination of avuncular relationship given two full-siblings.** A pedigree is shown in panel A that highlights a known avuncular relationship (individual 3) to two full-siblings (individuals 1 and 2). Panel B is a pediSNP image in which the avuncular individual is inserted as a pseudo-parent to both half-sibs for chromosome 7 with an output similar to the one in **Figure 6B**. A series of asterisks identify a region of opposite of inheritance (e.g. AA/BB alleles at a given locus in individuals 1 and 2). SNPduo images provide IBS observations between individuals 1 and 3 (panel C), 2 and 3 (panel D), and 1 and 2 (Panel E). Note that individuals 1 and 2 are both related to individual 3 in the boxed region but are unrelated to each other. Panel F provides an ideogram for chromosome 7.
(EPS)

Figure S11 **Determination of avuncular relationship of NA21617 to the half-siblings NA21312 and NA21370.** A pedigree is shown highlighting an inferred avuncular relationship (NA21617) to two half-siblings (panel A; NA21312 and NA21370). Panel B is a pediSNP image in which the avuncular individual’s genotype (NA21617) is compared to the genotypes of the half-siblings (NA21312 and NA21370) along chromosome 3. Note that the boxed region with asterisks highlights an opposite inheritance region. SNPduo images show the pairwise IBS observations between individuals NA21312 and NA21617 (panel C), NA21370 and NA21617 (panel D), and NA21312 and NA21370 (panel E). Note that individuals NA21312 and NA21370 are both related to individual NA21617 in the boxed region but are unrelated to each other. Panel F provides an ideogram for chromosome 3.
(EPS)

Supporting Information S1 **Assumptions and methods for reconstruction of relationships given genotype data.** A supporting document is attached that provides a method to reconstruct second-degree relationships (i.e. half-sibling and avuncular), third-degree relationships (i.e. first-cousin) and fourth-degree relationships based on patterns of sharing regions IBD. Important assumptions for this method are provided that details scenarios in which this method should be applied and outlines circumstances that suggest atypical relatedness is present that warrants a cautious interpretation. These methods were applied to the HapMap populations described in this paper. More specifically, this method was used to construct **Figure 3** and **Figure S3** within the MKK population.
(DOC)

Table S1 **IBD estimates for previously annotated and novel relationships.** We report the IBD estimates for every pairwise comparison that we report as related within HapMap Phase III release 3 (n = 2,261). This includes previously annotated relationships (denoted by column headers indicating presence in Pemberton et al. [19] or Kyriazopoulou-Panagiotopoulou et al. [20]. We provide the estimated relationship coefficient for pairs that we were able to reconstruct according to the methods. This list includes all relationships with a K1 greater than 0.025 (including ID/MZ that have K2 ∼1.0) as well as the relationships between the parents of inbred individuals.
(XLSX)

Table S2 **Regions of homozygosity by chromosome and position.** We report chromosome and position information for every region of homozygosity ≥2 Mb and containing ≥400 informative SNPs for every individual. Abbreviations used: Individual ID, represents each HapMap individual; Start, where homozygous region starts with SNP position provided; Stop, where homozygous region ends with SNP position provided; Size (Mb), size of region based on start and stop SNP positions; Number of SNPs, number of SNPs present in the region reported based on start and stop SNP positions; SNPs/Mb, the average number of SNPs per megabase found within the reported region. There are 3,457 rows in the table (listing all HapMap phase III individuals and regions), including 3,240 identified regions.
(XLSX)

Table S3 **Total amount of homozygosity per individual.** We report total amount of homozygosity in Mb for every individual based on the sum of regions present in a given individual as provided in Table S1. Abbreviations used: Individual ID, represents each HapMap individual; Total Mb, indicates the total length of all reported homozygous segments in megabases; Total SNPs, indicates the total number of SNPs present in all reported homozygous segments; Total regions, indicates the number of reportable homozygous regions within a given individual; Average size (Mb), indicates the average size of the reported regions for a given individual; Average SNPs, indicates the average number of SNPs present within a reported region. There are 1,397 entries (one per HapMap phase III individual).
(XLSX)

Table S4 **Summary of relationships that can be identified.** Given a degree of relationship, different types of relationship can be proven based on a given number of individuals and sharing schema. Abbreviations: 2°, second-degree relationships; 3°, third-degree relationships; 4°, fourth-degree relationships; # Inds, minimum number of individuals required; # Ped., the number of pedigrees that can result from the minimum number of individuals present that fit the sharing schema (these pedigrees are indistinguishable from each other); AV, avuncular/materteral; FC, first-cousin; GA, great-avuncular; GG, grandparent-grandchild; HS, half-sibling; IBD, identity-by-descent; Rel., relationship; Req. Rel., required relationship.
(XLSX)

Table S5 **Recommended K value thresholds for recently related individuals.** Given a degree of relationship, the K values are distributed around the theoretical expected value. These distributions can be estimated and used to infer a relationship. Certain K values are presented that highlight abnormal sharing in certain relationships. Abbreviations: Expected, expected K coefficient given the relationship type; Estimated K range (within 2SD), variation surrounding the expected K value based on known relationships; Abnormal K (outside 3SD), recommended K values that should be considered as abnormal (with caution) based on known relationships; R. degree of relationship (percent of genome shared), calculated as K2+(K1/2); N, number of relationships in the distribution; Source, indicates publication where data originated; ID/MZ, identical samples or monozygotic twins; ^, these values are recommendations and should only be applied when analyzing known relationships; *, is not 3 SD away from the mean for full-siblings but serves to maintain proper delineation of full-sibling from a second degree relationship with bilineal relatedness (e.g. double first-cousins); [6], indicates reference six within the supporting document (i.e. Stevens et al. 2012).
(XLSX)
