# Supplementary material for: Unexpected Relationships and Inbreeding in HapMap Phase III Populations
Source: PLoS One. 2012 Nov 19;7(11):e49575. doi: 10.1371/journal.pone.0049575 (PMC3501496; doi:10.1371/journal.pone.0049575)
Supplement: Supporting Information S1 — Assumptions and methods for reconstruction of relationships given genotype data. A supporting document is attached that provides a method to reconstruct second-degree relationships (i.e. half-sibling and avuncular), third-degree relationships (i.e. first-cousin) and fourth-degree relationships based on patterns of sharing regions IBD. Important assumptions for this method are provided that details scenarios in which this method should be applied and outlines circumstances that suggest atypical relatedness is present that warrants a cautious interpretation. These methods were applied to the HapMap populations described in this paper. More specifically, this method was used to construct Figure 3 and Figure S3 within the MKK population. (DOC) [file pone.0049575.s012.doc]

# Supplemental material for “Unexpected relationships and inbreeding in HapMap Phase III populations” by Eric L. Stevens, Joseph D. Baugher, Matthew D. Shirley, Laurence P. Frelin, Jonathan Pevsner.

# Outline

1. Method for reconstruction of pedigrees
   1. Overview
   2. Metachondromatosis genotype data
   3. Assumptions for pedigree reconstruction
   4. Second-degree relationship reconstruction
      1. Avuncular/Materteral (Scenarios 1 and 2)
      2. Half-sibling (Scenario 3)
   5. Interpretation of pedigrees involving a third or fourth-degree relationship (Scenario 4)
   6. Ruling out potential relationships (Scenario 5)
2. Validation and application of method
   1. Introduction of pediSNP software for visual confirmation
   2. Reconstruction of a known pedigree
   3. Application to HapMap

4. Web Resources

5. References

**1. Method for reconstruction of pedigrees**

# 1a. Overview

We present specific requirements useful for the reconstruction of previously unresolved relationships (e.g. resolving a second-degree relationship into half-sibling or avuncular/materteral) on the basis of visualized haplotype sharing patterns from at least three individuals. Previous work has shown the benefit that the addition of a third individual can be in resolving certain relationship types using both linked and unlinked markers . In particular, we unambiguously infer second, third, and fourth-degree relationships that meet specific requirements based on the estimated degree of relationship between the individuals in question. Inferring the degree of relationship for a given pair of individuals is provided by an IBD method, kcoeff , which estimates the percent of the genome shared IBD0 (K0), IBD1 (K1), and IBD2 (K2). This method is robust in the absence of parent-child relationships and uses information provided by single nucleotide polymorphisms (SNPs). This method relies on the presence (or absence) of specific patterns of allele sharing between these individuals over contiguous regions in the genome.

**1b. Metachondromatosis genotype data**

For a validation dataset, we obtained SNP genotype data from a published study that included 12 individuals of ‘known’ relationship in which the degree of relationships for all individuals has been verified . The expected coefficients of relatedness ranged from 1/2 (parent-child and full-sibling) to 1/32 (first-cousins who were twice-removed) and zero (unrelated). There were 66 pairwise comparisons involving all individuals in the pedigree.

**1c. Assumptions** **for pedigree reconstruction**

This method requires at least three individuals used in one of two ways. First, one can establish the relationship type of a second or third-degree relationship between two individuals with reference to a third if certain patterns of sharing are met. Second, one can begin with two individuals of previously known relationship (e.g. full-siblings or half-siblings) and reconstruct their relationship to a third individual. The methods outlined below make the following assumptions by which specific patterns of chromosomal sharing are informative:

1. Parent-child (K0 of 0, K1 of 1, and K2 of 0) or full-sibling (K0 of ¼, K1 of ½, and K2 of ¼) relationships are unnecessary for these methods, with a single exception in which full-siblings are used to establish an avuncular relationship. We refer to K0, K1, and K2 as estimates of Cotterman’s coefficients of relatedness k0, k1, and k2 as inferred by kcoeff software .
2. All relationships have been estimated with kcoeff. The program obtains optimal estimates of K0, K1, and K2 when the window size used is approximately 150 SNPs per 500k total SNPs. For example, a window size of 450 should be used for datasets containing 1.5 million total SNPs. The more SNPs a window size contains, the harder it will be to assess accurate boundaries between IBD states that will lead to slightly inaccurate IBD estimates. The fewer SNPs that a window size contains, the more IBD (especially IBD1) will be inferred because ancestral haplotypes will be picked up resulting in incorrect estimates of Cotterman coefficients that deal with recent relatedness. This program has been run using 360,000 to 1.5 million autosomal SNPs (some data not shown) . This program is not designed for data sets involving fewer than 100,000 SNPs. It is recommended that datasets involving more than 2 million SNPs be randomly pruned to within the suggested number of SNPs to avoid incorrect IBD values.
3. A K1 value above 0.35 but below that of parent-child relationships will be considered as a second-degree relationship. K1 values between second-degree relatives generally have a value of 0.5, with typical ranges of 0.3-0.7 due to variation in inheritance. Second-degree relatives who share more than expected (e.g. K1 ~0.70) are easily distinguished from parent-child (having K1 ~1.0) or full siblings (who have a similar K1~0.5 but who also have K2 ~0.25). K1 values of second-degree pairs have been observed as low as 0.30 but overlap with third-degree relationships (e.g. first-cousins) who have K1 values centered on 0.25 but as high as 0.35. Based on previous work, 97.5% of non-inbred, second-degree relationships have a K1 higher than 0.336 (n = 506) . Based on third-degree relationships from the present publication (assigned from second-degree relationships or higher – e.g. a child of a parent who is in a second-degree relationship to another individual would have a third-degree relationship to that individual) 97.5% had K1 values less than 0.334 (n = 162). Thus, we conclude that a K1 threshold of 0.35 is useful for distinguishing second and third degree relationships, with the knowledge that gross outliers of either distribution may be misclassified. **Table S5** provides a range of Cotterman coefficients for regular (normal) relationships. Note that relationships including and more distant than third-degree relationships are not classified by kcoeff alone and must have other information present.
4. Patterns of chromosomal IBD sharing are used to confirm or rule out a specific relationship. A minimum region size of 10 Mb is appropriate for visual confirmation of the different sharing schemas (described below) with multiple regions providing additional support. In fact, detection of more than one region is required. For example, the scenario involving three individuals in which one of them is in an avuncular position to two half-siblings is expected to have 12.5% of the genome shared in a particular pattern (see below).
5. The data used to measure K0, K1, and K2 by kcoeff will be used to find informative patterns (allowing for a minimum marker density of over 120 SNPs per Mb – based on a minimum of 360,000 autosomal SNPs).
6. Unrelated individuals are classified in this supporting document as having no *recent* common ancestor and having a K1 value below 0.025 (below the theoretical value of second-cousins once-removed). A pair of individuals lacking recent relatedness will not have regions that would be inferred to be IBD based on visual analysis (i.e. regions that span more than 10 Mb and have a lack of IBS0 calls).
7. This method was developed to assist with the reconstruction of human pedigrees. Extension to other species would require species-specific estimates of K0, K1, and K2 distributions for annotation of relationship types.
8. All individuals are assumed to be non-inbred. Individuals with atypical genome-wide homozygosity levels (F value greater than 1/128) should be analyzed cautiously because atypical relatedness (recent inbreeding) is present that could affect the application of this approach.
9. Finally, pairwise comparisons with unexpected estimated K0, K1, and K2 estimates as outlined in **Table S5** should be interpreted with caution as there is evidence that an atypical relationship exists. For example, a pairwise comparison with a K1 value of 0.40 and a K2 value of 0.04 is expected for double first-cousins; however, this could also be a second-degree relationship with additional bilineal relatedness. These relationships could affect the application of this method for pedigree reconstruction unless other supporting information is available.

**1d. Second-degree relationship reconstruction**

**1d i. Establishment of avuncular/materteral relationships (Scenarios 1 and 2)**

If three individuals are in second-degree relationships (**Figure S5A**), we can posit that two of these individuals must be half-siblings (**Figure S5A**, individuals 1 and 2) (Scenario 1) and apply the following schema to establish which individual (if any) is in the avuncular/materteral position and which individuals are the half-siblings (Scenario 1). This method can also be used to define the individual in the avuncular/materteral position (**Figure S5B**; individual 3) if the other individuals are full-siblings (**Figure S5B**; individuals 1 and 2) (Scenario 2). In these scenarios, avuncular/materteral relationships can be established using the following rationale:

1. Individual 3 will share approximately 25% of his/her genome IBD2 with the parent of individuals 1 and 2, because they are full-siblings.
2. The genotypes of individual 3 can be interpreted as corresponding to the parent’s genotypes when inspecting the patterns by which alleles are transmitted to individuals 1 and 2 within this region of IBD2.
3. In such regions, there is an opportunity for individual 3 to share a region IBD1 with each child, without IBD sharing between the siblings. Since a parent transmits a single allele to his/her child, a pair of siblings can either inherit the same allele (identical inheritance) or a different allele (opposite inheritance).
4. This sharing schema is expected to cover 12.5% of the genome (because individual 3 shares 25% of his/her genome IBD2 with the parents of individuals 1 and 2). Full-siblings may also share a region IBD1 inherited from the other parent (i.e. not the full-sibling of individual 3).

This method can also rule out avuncular relationships if there are two full-siblings who are related to a third individual in a second-degree manner that does not produce this opposite inheritance schema. If it is not known which two out of the three (all related in a second-degree manner) are half-siblings, one can place each individual into a potential avuncular position to determine if the above sharing schema is present in any of the three available configurations. If none of the three configurations produces this sharing, then one can be certain that there are two half-siblings (although their identities are unknown) and a third individual (again unknown) who is either a grandparent or another half-sibling.

**1d ii. Establishment of half-sibling** **relationships (Scenario 3)**

If a pair of individuals share a second-degree relationship (individuals 1 and 2), and each is uniquely related to another individual (individuals 3 and 4 respectively), then individuals 1 and 2 must be half-siblings. They are related to each other through their common parent and to individuals 3 and 4 through their exclusive parents. This method can prove that two individuals are in fact half-siblings, only if there are two additional individuals (i.e. 3 and 4) present that fit the above criteria. Failure to establish the above schema cannot be used to rule out a half-sibling relationship. It is recommended that the relatedness between individuals 1 and 3 and between individuals 2 and 4 have a K1 exceeding 0.20. This allows for a minimum theoretical expected K1 of 0.05 between individuals 2 and 3 and between individuals 1 and 4 *if* individuals 3 and 4 are related to both individuals 1 and 2. In sum, you want to choose individuals that are close enough related to unique members of a potential half-sibling pair that a lack of relatedness between individuals 2 and 3 and between individuals 1 and 4 is due to them *being* unrelated and not because they are too distantly related to be detected by kcoeff.

**1e. Interpretation of pedigrees involving a third or fourth-degree relationship (Scenario 4)**

For three related individuals involving two second-degree relationships (K1 greater than 0.35) and an unknown degree of relationship (K1 below 0.35 and thus not able to be classified), a method is provided for distinguishing between third and fourth degree relationships. However, there are multiple possibilities as to the structure of the pedigree for each degree of relationship.

**Interpretation of pedigrees involving two second-degree and one third-degree relationship(s)**

Cases in which the unknown relationship is third-degree can be confirmed according to the schema below and are illustrated in **Figure S6A-E**.

1. Individual 1 will share regions IBD with individual 2 independent of individual 3 (**Figure S6A**; see orange haplotype).
2. Individual 3 will share regions IBD with individual 2 independent of individual 1 (**Figure S6A**; see orange haplotype).
3. Individual 1 will also share regions IBD with individual 3 independent of individual 2 (**Figure S6A**; see black haplotype.)
4. There are two pedigrees that would be indistinguishable from each other. The first (**Figure S6A**) includes a grandparent (individual 2) related to two grandchildren (individuals 1 and 3) who are first-cousins. The second (**Figure S6B**) occurs when a person (individual 2) is in an avuncular position to two first-cousins (individuals 1 and 3).
5. A third possibility (**Figure S6C**), but one that can be distinguished, occurs when one individual (individual 3) is in a grandparental position to one individual (individual 2) and in a great avuncular position to another (individual 1) while individuals 1 and 2 are also half-siblings. In this case, individuals 1 and 2 would have regions shared IBD2 since they would have first-cousin plus half-sibling relatedness. The K1 of these individuals would be higher than expected for second-degree relatedness (although K1 would not approach 1). The presence of IBD2 provides more evidence for reconstructing such a pedigree.
6. A fourth possibility (**Figure S6D**) exists in which individual 1 is the half-sibling of individual of 2 who is the half-sibling of individual 3 while individuals 1 and 3 are both first-cousins. This is different than the other pedigrees since it is not possible for them to all share the same haplotype, providing proof of this relationship type.
7. The final pedigree (**Figure S6E**) presents individual 1 as a half-sibling to individual 2 who is a half-sibling to individual 3 while individual 1 is a first-cousin to individuals 2 and 3. This pedigree can be distinguished from the ones above because individual 3 cannot share different haplotypes IBD with individuals 1 and 2 (i.e. opposite inheritance as is possible in **Figure S6C**) and individuals 1 and 2 will share regions IBD2.

This method works to prove that two of the individuals are first-cousins (individuals 1 and 3; see **Figure S6A, B, D**) or first-cousins plus half-siblings (individuals 1 and 2; see **Figure S6C, E**).

**Interpretation of pedigrees involving two second-degree and one fourth-degree relationship(s)**

Two pairs of individuals (**Figure S7A-E**; individuals 1/2 and 2/3, respectively) are labeled as having second-degree relationships and the degree of relationship between individuals 1 and 3 is undefined. The following scenario (**Figure S7A-E)** occurs when the regions shared between individuals 1 and 3 are dictated by the regions shared between individuals 2 and 3:

1. Individual 2 will share regions IBD with individual 3.
2. Individual 1 will also share regions IBD with individual 3 but can only share the same regions that individuals 2 and 3 share. This is illustrated by the yellow blocks representing the same haplotype in **Figure S7A** and the asterisk indicating the yellow block that supports this relationship type (and is shared by individuals 1, 2 and 3).
3. Individual 1 and individual 2 may share regions that neither shares with individual 3 (e.g. **Figure S7A**, red alleles).
4. Individual 2 and individual 3 may share regions that neither shares with individual 1 (e.g. **Figure S7A**, blue alleles).
5. Individual 2 must be a grandparent, but the placement of individuals 1 and 3 cannot be resolved within the pedigree. For **Figure S7A-E**, individuals 1 and 3 are interchangeable and are shown for illustrative purposes only.

This method works to prove that a person (individual 2) is a grandparent if there are two second-degree relationships among three individuals that fit the criteria above. Individuals 1 and 3 can be designated as a fourth-degree relationship but one cannot distinguish who is the grandchild of individual 2 and who is the relative of individual 2 unless there is more information such as ages or previous annotation. In total, there are 5 possible pedigrees (**Figure S7A-E**) given three related individuals involving two second-degree and one fourth-degree relationship.

**1f. Ruling out potential relationships (Scenario 5)**

The above methods (scenarios 1-4) can be used alone or in multiple combinations to rule out potential relationship types. This rationale is used on a case-by-case basis. For example, **Table 3** highlights NA21300 compared to both NA21520 and NA21613 in an inferred half-sibling relationship. Scenario 5 involves the combination of the lack of Scenario 2 (to confirm a potential avuncular relationship) and the lack of Scenario 4 (to potentially confirm a grandparent-grandchild relationship). Scenario 3 was used to confirm a half-sibling status as they were both related to other unique individuals.

**2. Validation and application of method**

**2a. Introduction of pediSNP software for visual confirmation**

This program identifies informative patterns of SNP genotypes in trios of individuals . The pediSNP program was initially designed for the analysis of individuals from a nuclear family unit (i.e. full-siblings compared to a parent). We now extend this same software program to analyze relatedness involving any three members of a pedigree. Consider the pattern AA/BB/AB (i.e. individual 1 [pseudo-child1], individual 2 [pseudo-child2], and individual 3 [pseudo-parent]). In that scenario, individual 1 shares a region IBD1 with individual 3 in the same chromosomal region that individual 2 shares a region IBD1 with individual 3. This is recorded as a string of black dots in the opposite inheritance (“OPP”) track for tens of megabases. For informative tracks to provide support for a given relationship, regions should be > 10 Mb that are solely one color (e.g. black, individual 3 shares with individual 1 and 2 with 1 and 2 being unrelated at that locus: AA/BB/AB). The web-based program on the author’s website requires two parents to run. The choice of the second pseudo-parent is arbitrary since the output detailing the comparisons to the pseudo-children can be ignored.

## 2b. Reconstruction of a known pedigree

We applied these methods to a five generation pedigree to demonstrate its ability to confirm or provide evidence for known relationships . We had previously run kcoeff, a program that estimates Cotterman coefficients of relatedness K0, K1, and K2, on this pedigree for every pairwise comparison to confirm their degree of relationship . We used the output of SNPduo, which plots the IBS observations between a pair of individuals along a chromosome , and pediSNP, which plots informative classes from comparisons based on genotypes from three individuals , to visualize the various sharing schemas. We then classified the relationship type by applying our pedigree reconstruction methods.

We analyzed three individuals from whom we could possibly support a grandparent-grandchild relationship (**Figure S8A**; Scenario 4). Our assumption for this relationship is that the sharing between a grandparent and a relative will dictate the sharing between the relative and the grandchild since the relatedness comes through the grandparent. In this example, we had a grandchild (individual 1), a grandparent (individual 2) and an uncle to the grandparent (individual 3). We visualized IBS sharing between the grandparent and the uncle (**Figure S8B**) and between the grandchild to his great-great-uncle (**Figure S8C**) along chromosome 7 (**Figure S8D**) using SNPduo software. As expected, both IBS analyses showed an absence of IBS0 (inferred presence of IBD1) between the grandchild and the great-great-uncle only in the regions that were shared between the grandparent and the uncle (**Figure S8B, C**; see regions 1-3). **Figure S8B** also shows extensive, additional regions of inferred IBD1 between the grandparent and the uncle not shared with the grandchild (**Figure S8C**), which is allowable because the grandfather did not transmit those alleles to the grandchild. This method only confirmed that individual 2 was the grandparent and provided evidence supporting the known relationships (e.g. individual 1 and 3 have a fourth-degree relationship). Since there was no evidence against individual 2 being a grandparent (e.g. individuals 1 and 3 share regions not shared between 2 and 3), there was no indication of an error in the identities of 1 and 3.

We applied a method of identifying regions of opposite inheritance between two known half-brothers (**Figure S9A**; individuals 1 and 2; Scenario 1) when compared to their uncle (individual 3) to prove an avuncular relationship (if present) given three individuals who are all second-degree relatives. We used outputs generated from the pediSNP program (**Figure S9B**) to visualize the inheritance patterns. The presence of a region of opposite inheritance proves that individual 3 is in an avuncular position to the half-siblings 1 and 2 since individual 3 shares a region of IBD with both individuals 1 and 2 (who are unrelated at this locus).

We complemented this analysis with SNPduo (**Figure S9C-E**) to visualize regions of IBS0 that were consistent with opposite inheritance (**Figure S9B**). We observed a locus greater than 10 megabases (see boxed region with asterisks) in which the uncle shared IBD1 with both individuals (**Figure S9C, D**) but the half-siblings were IBD0 at the same position (**Figure S9E**) along chromosome 7 (**Figure S9F**). This sharing schema confirms the presence of an avuncular relationship to two half-siblings.

This same sharing schema can also confirm an avuncular relationship (**Figure S10A**; individual 3) to two full-siblings (individuals 1 and 2; Scenario 2). There is a region outlining the outputs from pediSNP (**Figure S10B**) and SNPduo **(Figure S10C-E**) along chromosome 7 (**Figure S10F**) in which there is an opposite inheritance segment that is consistent with the occurrence of an avuncular relationship (see boxed region with asterisks).

**2c. Application to HapMap**

We applied these methods to the MKK population and present part of the reconstructed pedigree in **Figure 3** and a more detailed version in **Figure S3**. An example of these methods (Scenario 1) is applied in **Figure S11A-F** in which NA21617 is inferred to be the aunt of NA21312 and NA21370 because she shares a region IBD1 with both individuals at the same chromosomal position (**Figure S11B-D)**; however, NA21312 and NA21370 are unrelated to each other at that locus (**Figure S11E)**.

NA21351 (individual 1), NA21352 (individual 3), and NA21414 (individual 2) are members of MKK from **Figure 3** that illustrate numerous sharing mechanisms to place NA21351 and NA21352 as first cousins with NA21414 as their uncle (Scenario 3). NA21414 is inferred to be in an avuncular position to the first-cousins NA21351 and NA21352 because tracts of opposite inheritance were detected in which NA21414 shared a region IBD1 with both NA21351/NA21352 at the same chromosomal location and NA21351 and NA21352 were unrelated to each other at this position. NA21414 was ruled out as being a grandparent to NA21351/NA21352 (who would also have regions of opposite inheritance with the first cousins) because of the relationship to NA21303 in which NA21351 and NA21352 shared regions with NA21303 independent of NA21414 (data not shown; Scenario 3 and 5). Note that we assumed a K1 between 0.20 and 0.35 as being third-degree for the illustrative purposes of reconstructing multiple clusters into a single pedigree within **Figure S3** (indicated by *).

A summary of the relationships that can be identified from the methods established above is presented in **Table S4**.

**4. Web Resources**

Pevsner lab website (for kcoeff, SNPduo, and pediSNP): http://pevsnerlab.kennedykrieger.org/.

**5. References**

1. Sieberts SK, Wijsman EM, Thompson EA (2002) Relationship inference from trios of individuals, in the presence of typing error. American Journal of Human Genetics 70: 170-180.

2. Wang J (2007) Parentage and sibship exclusions: higher statistical power with more family members. Heredity (Edinb) 99: 205-217.

3. Stevens EL, Heckenberg G, Roberson ED, Baugher JD, Downey TJ, et al. (2011) Inference of relationships in population data using identity-by-descent and identity-by-state. Plos Genetics 7: e1002287.

4. Sobreira NL, Cirulli ET, Avramopoulos D, Wohler E, Oswald GL, et al. (2010) Whole-genome sequencing of a single proband together with linkage analysis identifies a Mendelian disease gene. PLoS Genet 6: e1000991.

5. Conrad DF, Pinto D, Redon R, Feuk L, Gokcumen O, et al. (2010) Origins and functional impact of copy number variation in the human genome. Nature 464: 704-712.

6. Stevens EL, Heckenberg G, Baugher JD, Roberson ED, Downey TJ, et al. (2012) Consanguinity in Centre d'Etude du Polymorphisme Humain (CEPH) pedigrees. Eur J Hum Genet.

7. Ting JC, Roberson ED, Currier DG, Pevsner J (2009) Locations and patterns of meiotic recombination in two-generation pedigrees. BMC Med Genet 10: 93.

8. Roberson ED, Pevsner J (2009) Visualization of shared genomic regions and meiotic recombination in high-density SNP data. PLoS One 4: e6711.
